# Intervention strategies to prevent mental health problems and improve resilience in employed parents from conception until the child is 5 years of age: a scoping review

**DOI:** 10.1186/s12884-024-07043-4

**Published:** 2025-01-09

**Authors:** Neeltje Crombag, Bieke Bollen, Eline Vancoppenolle, Thomas Vandendriessche, Dagmar Versmissen, Martha Paisi, Jill Shawe, Susan Garthus-Niegel, Annick Bogaerts

**Affiliations:** 1 REALIFE Research Group, Women and Child, Department of Development and Regeneration, KU Leuven, Louvain, 3000 Belgium; 2 L-C&Y, KU Leuven Child & Youth Institute, Louvain, 3000 Belgium; 3 Department of Woman and Baby, University Medical Center Utrecht, Utrecht, the Netherlands; 4 KU Louvain Libraries 2Bergen – Learning Centre Désiré Collen, UZ Herestraat 49, P.O. Box 411, Leuven, 3000 Belgium; 5 Thomas More University of Applied Sciences, Antwerp, Belgium; 6 School of Nursing and Midwifery, University of Plymouth, Plymouth, UK; 7 Royal Cornwall Hospitals NHS Trust, Truro, Cornwall UK; 8 Institute and Policlinic of Occupational and Social Medicine, Faculty of Medicine, Technische Universität Dresden, Dresden, Germany; 9 Institute for Systems Medicine (ISM), Faculty of Medicine, Medical School Hamburg MSH, Hamburg, Germany; 10 Department of Childhood and Families, Norwegian Institute of Public Health, Oslo, Norway

**Keywords:** Working pregnant parents, Scoping review, Perinatal, Social support, Mental health problems, Resilience

## Abstract

**Aim:**

To understand the extent and type of evidence in relation to the effectiveness of intervention strategies targeting working pregnant women, and their partners, for the prevention of mental health problems (depression, anxiety) and improving resilience, from conception until the child is 5 years of age.

**Methods:**

A scoping review was conducted searching Pubmed (including Medline), Embase, Web of Science Core Collection and Scopus. Inclusion criteria were based on population (employed parents), context (from -9 months to 5 years postpartum) and concept (mental health problems, resilience and prevention/ preventative interventions).

**Results:**

Of the 17,699 papers screened, 3 full text papers were included. Studies focused on intervention strategies for working parents which showed a relationship with a reduction in mental health problems (depression and/or anxiety). The intervention strategies extracted from the literature referred to ‘social support’. Social support provided by both the social and the work environment correlated with prenatal stress and depressive symptoms in the postpartum period, and supports a healthy work-family balance.

**Conclusion:**

Social support seems to have a positive association with the reduction of mental health problems. However, there are still important gaps in the literature such as a lack of RCT designs to test effectiveness of interventions and systematic reviews. Findings from this study may provide a roadmap for future research to close these gaps in knowledge.

**Supplementary Information:**

The online version contains supplementary material available at 10.1186/s12884-024-07043-4.

## Introduction

The perinatal period, encompassing pregnancy and the first year after birth, is characterized by major psychological, behavioral, social, and biological changes for (expectant) mothers in a short period of time [1–5]. According to the World Health Organization, for one in five women, the perinatal period is overshadowed by mental health problems [6]. Perinatal mood and anxiety disorders (PMADs) is an overarching term that describes mental health disorders during this period [5]. The most known and researched type of PMADs is postpartum depression. A meta-analysis by O’Hara et al. [7] on postpartum depression found an overall prevalence of 13% within the first 12 weeks following birth (*n* = 12,810 95% confidence interval 12.3% to 13.4%). A more recent systematic review from 2005 including 28 studies provides a prevalence ranging from 6.5% to 12.9% at different time points (both pre- and postnatal) with a pooled prevalence of depression to be 14% [8]. Two large recent meta-analyses show a high prevalence of postpartum depression with a pooled prevalence of 14% (with variations from 5.0% to 26.3% in different countries) [9], and show that women in low and middle-income countries are at particular high risk with depression reported in 24.7% of women in the first year postpartum [10]. In addition, partners are also at increased risk of depression during the perinatal period [11–13]. When specifically assessing the prevalence of perinatal mood disorders in both parents, a recent systematic review and meta-analysis found that in 3.18% of couples, both parents may concurrently experience perinatal depression [14].

For anxiety disorder and anxiety symptoms a systematic review and meta-analysis by Dennis et al. [15] found an overall prevalence for a clinical diagnosis of any anxiety disorder during pregnancy of 15.2% (95% CI 9.0–21.4) and 4.1% (95% CI 1.9–6.2) for a generalized anxiety disorder. Postnatally this was 9.9% (95% CI 6.1–13.8) for any anxiety disorder, and 5.7% (95% CI 2.3–9.2) for a generalized anxiety disorder [15]. Despite the variety in published prevalence, we can conclude that PMADs are common. In addition, the burden of PMADs on parents, experiencing these problems during the perinatal period may adversely affect the developing parent-infant relationship and infant’s early brain and regulatory maturation [16]. Prevention of PMADs therefore may positively impact the expectant and new parents, and their family.

Earlier reviews have indicated that preventative interventions targeted at women at risk for depression in the perinatal period are effective in the prevention of depression [17, 18]. Less is known about the effectiveness of interventions on other disorders such as anxiety and distress [19, 20]. For women without an a priori risk for developing PMADs, a recent systematic review and meta-analysis and investigated the effectiveness of universal intervention components such as psycho-education, mindfulness and cognitive behavioral therapy, on the prevention of symptoms of depression, anxiety and distress during pregnancy. This study indicated that those preventative interventions, as compared to no intervention, are effective in decreasing maternal depression in a low-risk population [21]. The effect on maternal anxiety and stress was less evident, likely due to the smaller number of studies available. This finding was supported by another systematic review focused on preventing anxiety and stress in both parents during the first 1,000 days, including pregnancy and postpartum [22]. Cognitive-behavioral therapy methods, mindfulness practices, and parent–child interaction were among the interventions employed. Despite limited literature, the available evidence suggests that cost-effective outcomes can be achieved by investing in preventive interventions during the perinatal period [23].

An important aspect of (expectant) parents’ daily life that is not taken into account as a variable in the above mentioned reviews is the parents’ work life. Data show that in 2019, 71% of mothers (with children between 0–14 years old) in Economic Co-operation and Development (OECD) countries were employed, with differences between countries ranging from 30 to 85% (www.oecd.org/els/family/database.htm). Becoming a parent can be considered a major life event, and comes with a shift in household composition and family responsibilities. Employed mothers have to juggle multiple roles (worker, partner, and mother) while physically recovering from childbirth [24]. For both parents, this involves transitioning to offering infant care 24/7 and adjusting to their ‘new normal’ while simultaneously readjusting to their roles as workers [25]. Experiencing an imbalance between parental demands and resources may trigger stress [26], placing an additional risk for developing mental health problems [27]. Furthermore, work-related factors, such as high psychological work demands, perceived lack of control over work and family, as well as a higher total workload and lower job flexibility have been associated with depressive symptoms in the postpartum period [28–30]. Psychosocial work stress and precarious working conditions may serve as additional risk factors for developing mental health problems [31].

The ability to cope with challenges, adversities and stress in life is generally understood as resilience [32]. Perinatal mental resilience in (expectant) employed parents may decrease the likelihood for parents to experience PMADs [33]. Despite the potential relevance, there are only few studies that explicitly examined factors that strengthen mental resilience and thus potentially reduce the negative effects of PMADs for parents. Moreover, literature specifically targeting preventative resilience factors for PMADs in employed parents is lacking.

Therefore, the objective of this scoping review is to identify the effectiveness of intervention strategies targeting the prevention of PMADs or improving resilience in *working* pregnant women, mothers, and their partners. Intervention strategies were defined as additional measures or programs designed to prevent PMADs in employed expectant and new parents. This scoping review extends the perinatal period from conception to age 5, a critical phase for infant and early childhood mental health. It therefore also accommodates differences in when parents return to work after childbirth, considering individual and cultural variations. In addition, intervention strategies in relation to work-related factors will be examined.

## Methods

A scoping review was conducted to map evidence regarding the type of intervention strategies targeting the prevention of PMADs and improving resilience specifically among employed pregnant women, mothers and their partners, until the child is 5 years of age. A scoping review is a preliminary assessment of potential size and scope of available research literature and aims to identify nature and extent of research evidence (usually including ongoing research). It serves as a comprehensive overview and can inform future systematic reviews [34–36].

### Protocol and registration

The scoping review protocol was developed in accordance with The Joanna Briggs Institute (JBI) methodology for scoping reviews [37] and registered with the Open Science Framework on 12/05/2022 (https://osf.io/n2wjz/).

### Eligibility criteria

The basis on which sources were considered for inclusion were population, concept, and context relevant for the study objective [37] (Table 1). Original and review articles were included on intervention strategies targeting the prevention of PMADs or improving resilience specifically *in working* pregnant women, mothers, and their partners, until the child is 5 years of age. Intervention strategies were considered as supplementary measures or programs available to working parents, in addition to the existing governance policies such as parental leave. For language, only English, Dutch, or French articles were included. No date or geographical limits were applied.

**Table 1:** Eligibility criteria

**Study characteristics**	*Inclusion criteria*	*Exclusion criteria*
*Population of interest*	*General population of working parents from -9 months to 5 years postpartum: Studies comprising employed pregnant women, mothers, and their partners*	*Studies comprising non-working parents* *Parents with pre-existing mental health problems*
*Context*	*Perinatal period, from -9 months (conception) extended until the child is five years of age*	*Childhood period* > *5 years of age*
*Concept*	*Perinatal mental health problems, resilience or prevention/ preventative interventions, interventions targeted at employed population*	
*Language*	*English, Dutch, or French*	
*Study type*	*(peer-reviewed) Original articles or review (Qualitative, quantitative or mixed methods)*	

### Information sources and search

For the search strategy a two-step approach was followed. In the first step, an exploratory search in PubMed (including Medline) using the concepts of ‘perinatal mental health’ and ‘interventions’ was undertaken to identify keywords and Mesh-terms to inform the second step. In this second step, all relevant terms were used to perform a comprehensive search in PubMed (including Medline -coverage 1946–2024) (see Appendix I, search) by two biomedical information specialists (TV; EV). Four concepts, “perinatal”, “parents”, “(mental) health”, and “employment”, were combined using the Boolean operator AND. Within each concept Mesh-terms and free text words (synonyms, relevant terms), to search in title, abstract, and keywords, were combined using OR. While we aimed at preventative interventions, it was decided to not include this as an additional concept in the search. Including "interventions" would have narrowed the search to papers explicitly using that term. To ensure a broader, unbiased search, we omitted the intervention concept to avoid limiting results to interventions termed as such. The systematic search strings used for Pubmed were adapted to the following databases: Embase (Embase.com—1974 to 2024), Web of Science Core Collection (SCI-EXPANDED – 1955 to 2024, SSCI – 1956 to 2024, AHCI – 1975 to 2024, CPCI-S – 1990 to 2024, CPCI-SSH – 1990 to 2024, BKCI-S – 2005 to 2024, BKCI-SSH – 2005 to 2024, ESCI – 2018 to 2024, CCR-EXPANDED – 1985 to 2024, IC—1993 to 2024), and Scopus (Scopus.com – 1788 to 2024). The comprehensive search was conducted on June 9th 2022, and an updated search was conducted on September 25th 2024 by a biomedical information specialists (EV). Following the search, all retrieved references from the databases were imported into Endnote (EndNote X9.3.3 /2013, Clarivate Analytics, Philadelphia, PA USA) and duplicates were removed according to the adapted method of Bramer et al. [38]. In addition to the search strings ran in the databases, the reference lists from all included articles were screened for additional studies (backwards and forward snowballing).

### Selection of sources of evidence

After uploading the deduplicated records into the screening application Rayyan, titles and abstracts were blind screened by two independent reviewers (NC and BB) for assessment against the eligibility criteria [39]. Potentially relevant records were retrieved in full. The full text of these selected records was assessed in detail against the eligibility criteria by three independent reviewers (NC, BB, and DV). Any disagreements that arose between the reviewers at each stage of the selection process were resolved through discussion, or with an additional reviewer (AB).

### Data charting process

Data from the included articles were charted in an extraction grid. The draft data extraction grid was modified and revised as necessary during the process of extracting data from each included evidence source. The data extracted included specific details about 1) general presentation (first author, year of publication, country of origin, aim, sample size, target population, perinatal period), 2) study design, 3) intervention/ intervention strategy (period, duration), 4) outcome evaluation (employment, mental health, and effect).

## Results

### Search results

The search strategy yielded a total of 34,610 articles (initial search 24.889 and update 9721). After removing duplicates, a total of 17,699 (initial search 12.591 and update 5108) were screened on title and abstract. The results of the search and the study inclusion process are depicted in a PRISMA flow diagram [40] (Appendix II PRISMA-ScR). Based on title and abstract screening, we were unable to identify any (randomized) controlled trials or systematic reviews regarding effectiveness of interventions for the prevention PMADs or improving resilience *specifically targeting employed parents*. However, we were able to identify scientific literature on intervention strategies to reduce PMADs, based on the eligibility criteria. Intervention strategies were sources of support available within the social or working context of working pregnant women, mothers, and their partners.

**Fig. 1:**
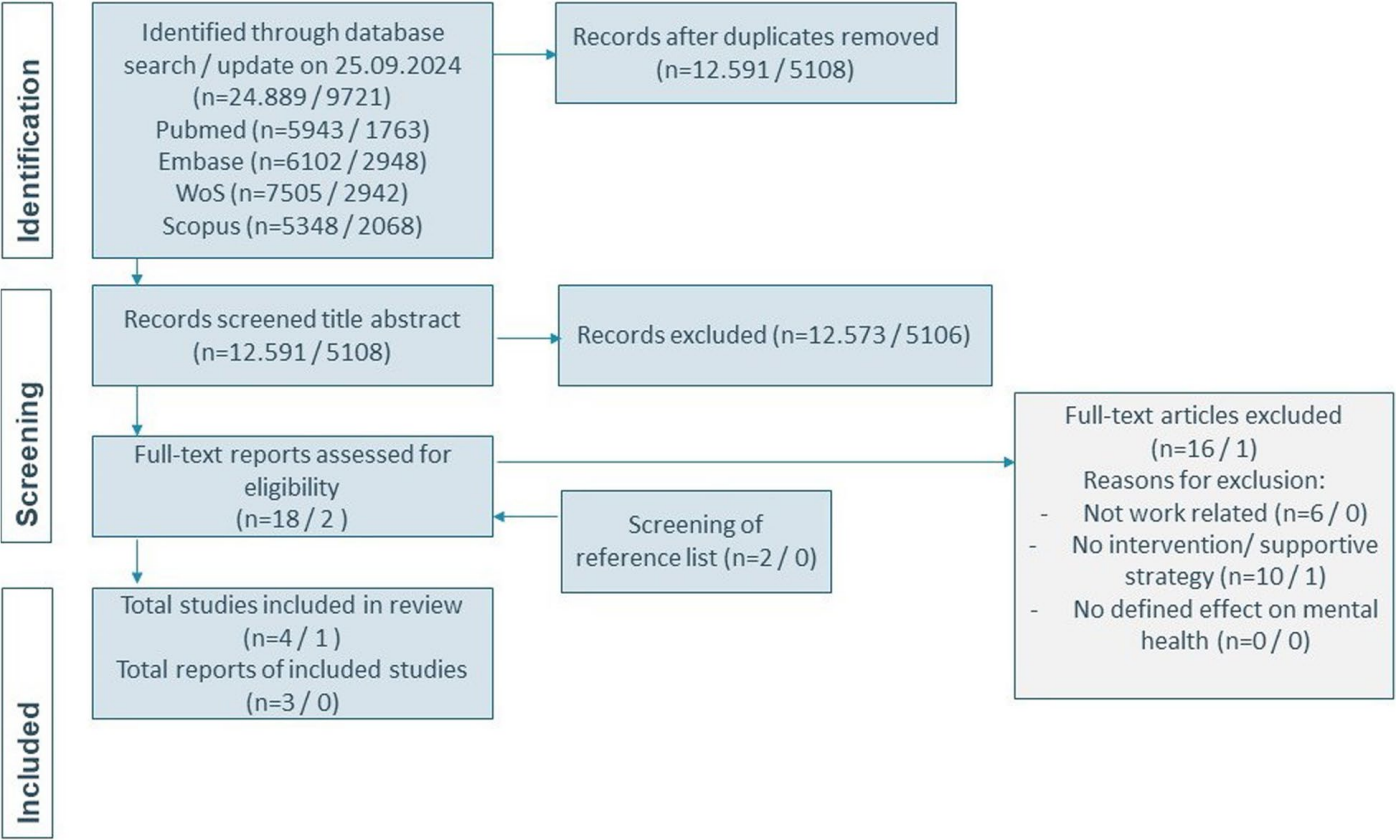
PRISMA flowchart of the study selection process; ‘*n* = /’ stands for the number of records retrieved from the first search and from the updated search

This resulted in 22 full-texts to be assessed for eligibility, of which 3 studies fulfilled the inclusion criteria. In addition to the previous findings, two studies on preventative interventions for perinatal mental health in working parents were identified: a randomized controlled trial protocol and a study on developing an intervention to support parents in the workplace (Fig. 1). These studies will be discussed in the results section but are not included in Table 2.

**Table 2:** Included studies and characteristics, encompassing studied interventions targeting working pregnant women, and their partners and the relation with outcomes

	**Author/Year of publication**	**Country of origin**	**Study design**	**Aim**	**Population and sample size**	**Intervention/ interventionstrategy**	**Duration intervention/ intervention strategy and period**	**Outcome employment**	**Outcome mental health**	**Outcome child related**	**Effect**
1	**Killien 2005** [41]	USA	Longitudinal survey study	To explore the role of social support in facilitating women’s return to employment during the 1st year postpartum.	Postpartum women, with partner, employed first child (employed at least 20 h per week) (*n* = 94)	Social support (both emotional support and practical help)	Not specified, postnatal	Satisfaction with decision to return to work, role performance, work-family balance.	--	--	➔Child care positive relation with return-to-work➔ Work support positive with work-family-balance at 12 m pp
2	**Gjerdingen 2014 **[42]	USA	Survey study	To characterize the relationship between employment and maternal depressive symptoms and whether it is mediated by social support	Postpartum women who gave birth in 2005 and completed two surveys in the ‘Listening to Mothers’ series (*n* = 700)	Social support	Not specified, postpartum	--	Depression	--	➔Maternal employment and strong social support, particularly non-partner support fewer depressive symptoms
3	**Jones 2022 **[43]	USA	Longitudinal survey study	The extent to which both maternal psychological and physical health are influenced by social support received at work during pregnancy	Pregnant employees and employees with children (*n* = 118).	Social support at work (Co-worker support; Supervisor support Stress during pregnancy)	Not specified, prenatal	Return to work	Prenatal stress; Postpartum depression; Recovery time from birth-related injuries	--	➔Supportive coworkers *and* supportive supervisors lower levels prenatal stress, postpartum depression and quicker recovery from birth

### Study characteristics

The included studies were published between 2004–2022, and all used a quantitative design, utilizing survey methodology (*n* = 3). The studies were conducted in one country: USA (*n* = 3). The selected articles described one intervention strategy: ‘social support’ (*n* = 3)). One study was conducted during the prenatal period, and 2 studies were conducted during the postnatal period. A summary of findings (interventions targeting working pregnant women, their partners and the relation with outcomes) are presented in Table 2.

### Social support

Three studies covered social support. More specifically, the articles focused on the role of social support in facilitating women’s successful return to and maintenance of employment during the first postpartum year [41]. One study focused on the mediating role of social support between employment and depressive symptoms [42]. The most recent study investigated whether both maternal psychological and physical health were influenced by social support received at work during pregnancy [43]. The findings of these three studies will be presented in more detail in the following paragraphs.

### Definition

**Fig. 2:**
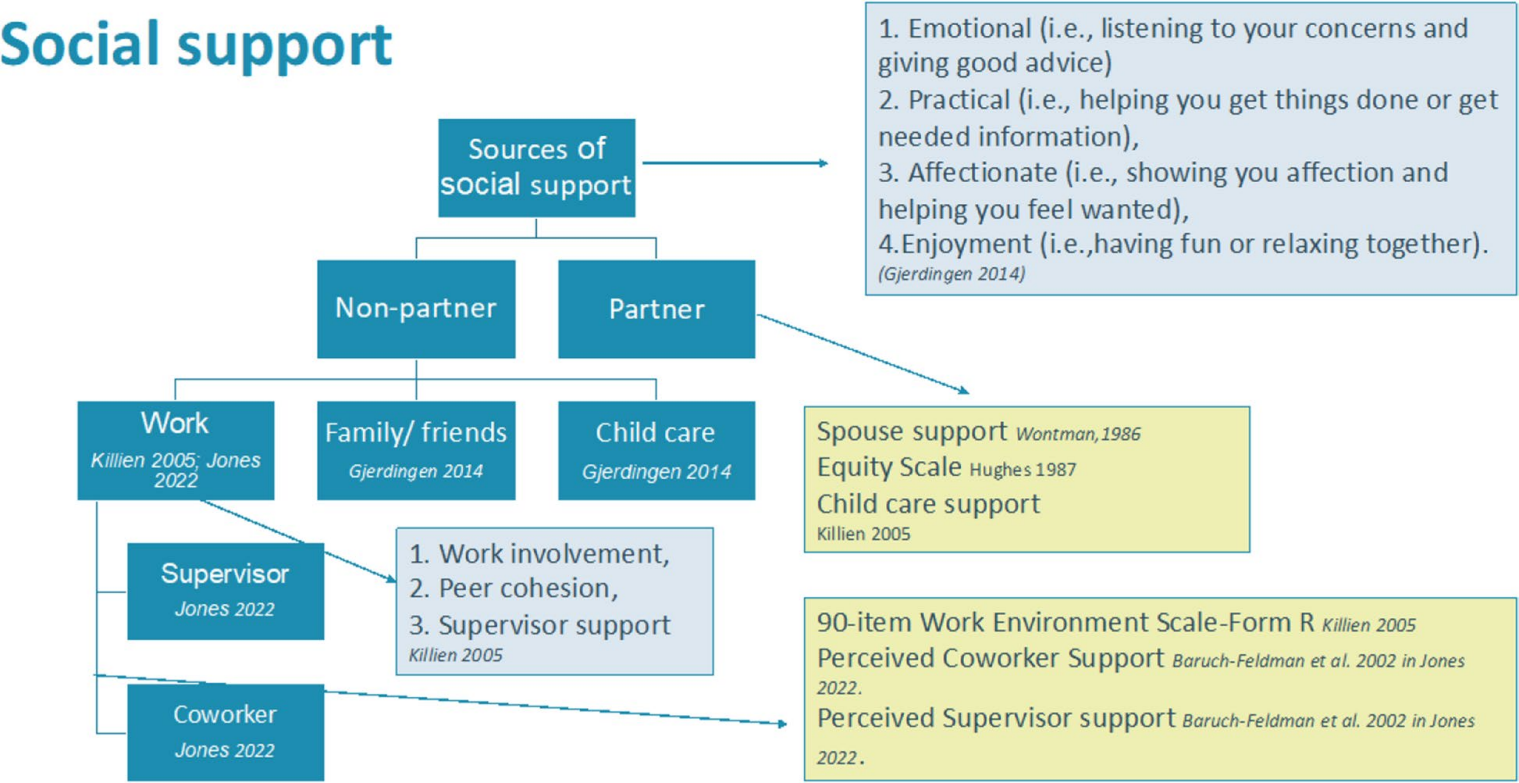
Social support of employed mothers, sources (dark blue), used measures (yellow) and dimensions (grey)

In the included studies social support was defined as emotional and practical support enabling postpartum employment [41]. More specifically, Gjerdingen et al. consider four dimensions of social support: emotional (e.g. listening to your concerns and giving helpful advice), practical (e.g. helping you get things done or get needed information), affectionate support (e.g. showing you affection and helping you feel wanted), as well as enjoyment (e.g. having fun or relaxing time together) [42]. Jones et al. do not provide a definition, but focused specifically on two sources of work-related social-support: co-workers and supervisor support [43]. Based on the included articles, Fig. 2 summarizes how social support can be understood in terms of its sources, used measures, and dimensions.

### Sources of support

The primary source of support, among women with a partner, was the partner. Partner support when returning to work, additional parental leave for the partner, and sharing household chores was considered helpful. While mothers were working, partners were considered as an important source of support regarding the care of the child, together with informal (family and friends) as well as more formal forms of childcare (e.g. nanny, day care, nursery school) [41].

Sources of social support at the workplace were either coworkers or supervisors. This was measured in terms of being caring, being interested, and supportive in getting the job done (coworker) as well as showing concern for the employees, giving them credit, and being caring (supervisor) [43]. The dimensions of support were described as ‘work involvement’, ‘peer cohesion’, and ‘supervisor support’ [41].

### Relationship with outcomes

As compared to unemployed women, postpartum employment and social support (from partner and others) were independently related with fewer depressive symptoms, even after controlling for demographic and health characteristics [42]. In addition, higher levels of coworker support in combination with higher levels of supervisor support during pregnancy seemed to positively impact prenatal stress and depressive symptoms in the postpartum period [43]. Social support within the work environment also played a positive role in achieving a more optimal work-family balance and improved role performance at 12 months postpartum [41]. Work-family balance refers to the extent to which a person’s job makes it difficult to coordinate work and family roles, while role-performance was defined as the extent to which a person perceives to be able to perform daily (work) activities [41]. When returning to work, a better work-family balance was positively related to having a partner who contributed significantly in household chores and family work [41].

### Preliminary studies on preventative strategies for perinatal mental health in working parents

Our search identified one protocol and one study on preventative strategies for perinatal mental health in working parents: a randomized controlled trial protocol and a study on developing an intervention to support parents in the workplace [44, 45].

The first study is a protocol on a randomized controlled trial testing of the MAternal Mental health in the WORKplace (MAMH@WORK) intervention. The protocol aims to promote maternal mental health from pregnancy through the first 12 months after delivery, improve the quality of mother–child interactions, and reduce perinatal absenteeism and presenteeism. The intervention adopts a cognitive-behavioral therapy-based psychoeducation approach through live group sessions, focusing on key skills such as recognizing signals and symptoms of mental disorders, reducing stigma, developing coping skills for stressful situations (including mindfulness training), enhancing help-seeking efficacy, and strengthening of emotional and cognitive self-regulation [45].

The second study focuses on the development of the Health in Planning, Pregnancy and Postpartum (HiPPP) Portal. This digital health intervention aims to promote healthy lifestyles and well-being for working women during the peripartum period. It employs key strategies, information, and support to enhance health and well-being, including assistance for returning to work after childbirth. Using Intervention Mapping methods, several content aspects for the portal were identified, including work safety, antenatal appointments, and pregnancy loss during pregnancy. For the postpartum period and return to work, the focus is on building confidence in the employee’s ability to return, providing breastfeeding support, and addressing postpartum depression [44].

## Discussion

We conducted a scoping review on interventions targeting working pregnant women, mothers, and partners to prevent PMADs or enhance resilience from conception to the child’s age of 5. Within the framework of this scoping review, we were unable to identify any (randomized) controlled trials or systematic reviews regarding interventions for the prevention of PMADs and improving resilience *specifically targeting employed parents*. This highlights the lack of controlled studies on effective interventions to prevent mental health issues in employed parents. However, we found literature on the intervention strategy “social support in the workplace,” which was associated with a reduction in PMADs, consistent with the review’s inclusion criteria. In addition to the previous findings, two studies on preventative interventions for perinatal mental health in working parents were identified: a randomized controlled trial protocol and a study on developing an intervention to support parents in the workplace [44, 45].

**Fig. 3:**
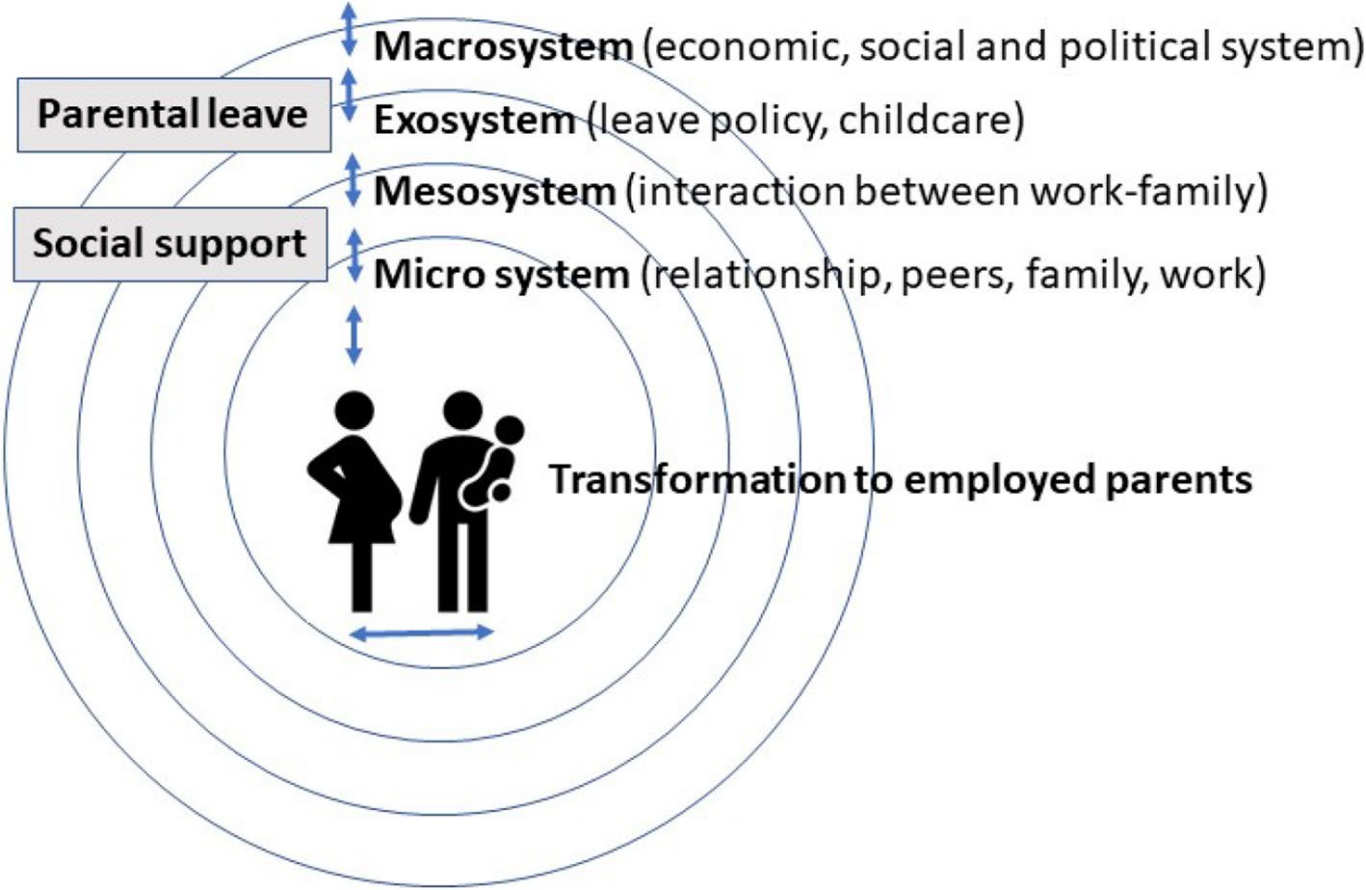
Bronfenbrenner model adapted for the transition into parenthood for employed parents [46]

The transition to parenthood of employed parents takes place in a context of interdependent systems. To understand systems that influence the parents’ mental health, the Bronfenbrenner model provides a helpful framework (Fig. 3). This framework comprises five interdependent systems: the micro system (family, parents, work), the mesosystem (the interaction between two or more aspects of the microsystem), the exosystem (social services, social structure, parental working conditions), the macrosystem (economic, social, and political systems), and the chronosystem (changes in individual or environment over the time) [46] (Fig. 3). At the micro-level, parents transform from employed individuals to employed individuals with children, which affects both their personal situation as well as their working situation.

In addition to their existing work roles and responsibility, new roles and responsibilities emerge. While it has been shown that working pregnant mothers seem to have lower levels of depressive symptoms than their non-working peers [47], the role transition is both physically and mentally challenging [24]. The transition takes place in a period in which parents are already at increased risk for developing PMADs, and a time in which first formative steps in bonding between parent and infant take place [15].

The interaction between both parents, their relationship, as well as direct support from their social network (family and friends) are proven intervention strategies that positively impact perinatal mental health. For perinatal depression, results from several systematic reviews and meta-analytic findings have consistently reported on the importance of social support. Social support is broadly defined as the provision of emotional (e.g. caring), or informational (e.g. notifying someone of important information) support, instrumental (e.g. helping with housekeeping), tangible (e.g. practical support like financial aid), and/or psychological support for somebody by the social network of family members, friends, or community members [47]. Having low levels of social support was significantly associated with prenatal depression and prenatal anxiety [48–50]. This relationship was also consistently seen in postpartum women [51–57].

While social support can be received from family and friends, as shown in this review, social support during pregnancy provided in the work-environment also has additional benefits for employed parents. Those effects can be seen both in the prenatal and the postnatal period. This support can come from co-workers, but its impact is greatest when supplemented by supervisor support [43]. In the workplace, raising awareness about the importance of this type of social support can be promoted through training initiatives [43]. In addition, interventions specifically including social support aspects, therefore may be a promising approach for reducing mental health problems in employed parents. Another form of work-related social support, might be the provision of high quality and reliable childcare at the workplace [42]. Especially during the sensitive period of transitioning back to work after childbirth, family-friendly work conditions may shape employed parents’ ability to be sensitive caretakers, ultimately benefiting children’s development [58]. This not only requires attitude change at the micro-level of the coworker/ supervisor, it also requires adjustment at the exosystem level. Social services that support expectant and new parents can only be achieved within a society that is supportive towards expectant and new parents and their children. On a macro level, sociocultural characteristics such as beliefs about and values related to parenthood shape those social rights, for example parental leave policies.

Parental leave is another form of macrosystem level support, offered through governance policies. Otto et al. compared the generosity of parental leave policies across European countries, highlighting how different policies affect the eligibility for, and duration and amount of such benefits individual parents are entitled to [59]. Their work also shows the large variability in public expenditure on maternity and parental leave [59].

**Fig. 4:**
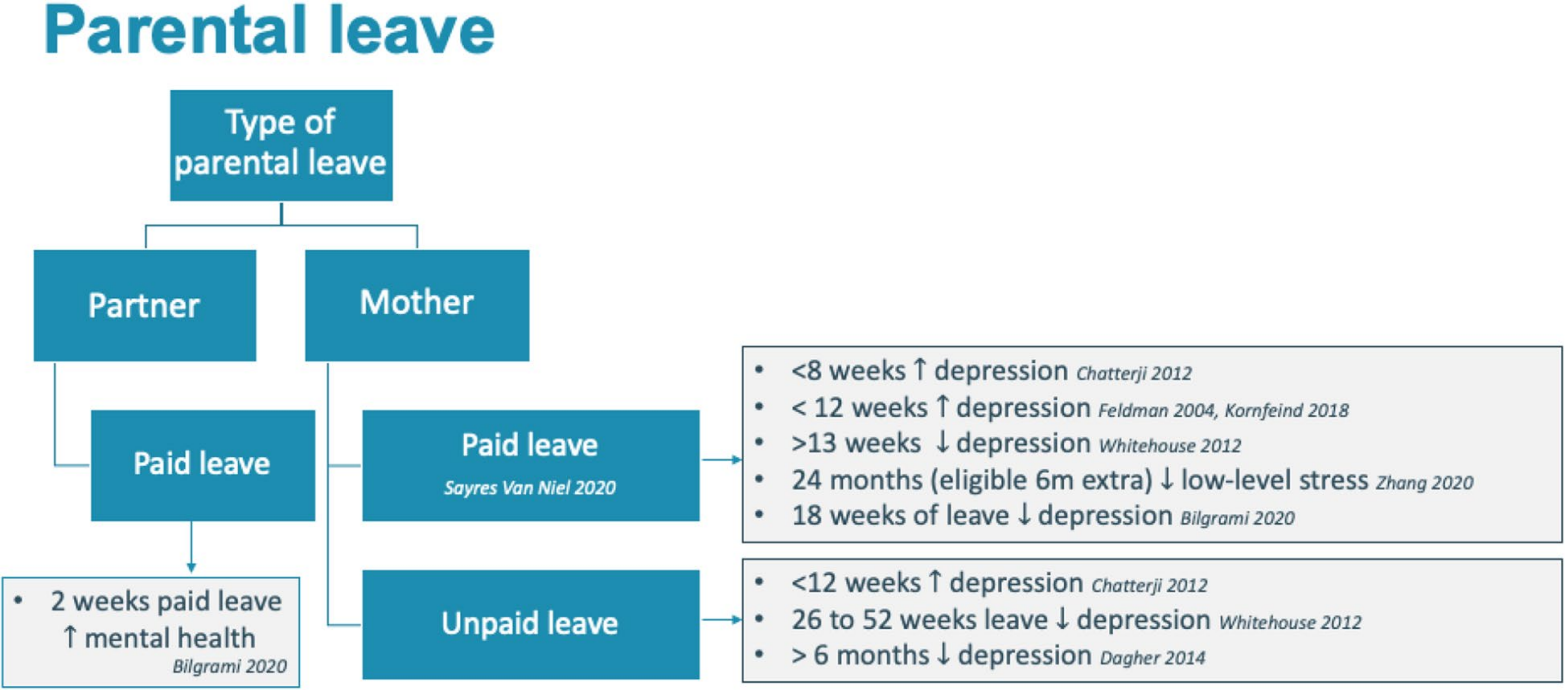
Leave type, duration, and payment

Several recent systematic reviews and a meta-analysis have shown that more generous leave policies, both in duration and payment, positively impact mental health. This includes reducing postpartum depression, improving mother–child interactions, enhancing maternal mental health (such as lowering depressive symptoms, psychological distress, and burnout), and decreasing the need for mental health care [60–64]. Such macro level system aspects have a cascading effect through all other levels and ultimately impact the micro-level of everyday life [59]. For example, maternity leave, of at least 12 weeks, has shown to have positive effects on parental mental health, with evidence supporting that additional leave up to 52 weeks further enhances mental health outcome in mothers [26, 65–68] (Fig. 4). Additional partner leave further enhances this effect [69]. The immediate impact of leave policies on perinatal mental health is one example of these cascading effects, shown from the intervention strategies found in this scoping review. As employed parents are part of a wider (macro) system, national and regional policies influence working conditions. For example, circumstances can vary in terms of having the possibility to work part-time, to be entitled to paid parental leave or having access to affordable child care [70]. On a more micro level, differences exist in the type of work, physical impact, emotional impact, but also the employees’ educational level, job satisfaction or burden play a role [30, 31].

In addition, the psychosocial work environment can affect parents’ health and wellbeing if there is a negative disbalance between work-efforts and low rewards [30, 31]. A recent German study found that such negative working conditions, for example, a sense of imbalance between effort reward, are a risk factor for developing symptoms of postpartum depression during maternity leave [31]. During the COVID-19 pandemic, a challenging time for working parents managing both work and childcare amid lockdowns, German studies found that work-privacy conflict and effort-reward imbalance were linked to depressive symptoms in parents [71]. Additionally, psychosocial work stress and work-privacy conflict were tied to weaker child-parent bonding [72, 73]. Resilience seems to have a protective role, buffering the association between work psychosocial stress and depressive symptoms [71].

Longitudinal data of employed parents across the transition to parenthood indicated that experiencing greater job autonomy and self-direction was related to less overreactive parenting and more involved parenting, leading to less behavior problems in their children. This indicates that work-experiences play an important role in shaping employed parents’ ability to be sensitive caretakers, ultimately benefiting children’s development [58, 74]. This potentially provides opportunities within the work-environment for additional preventative interventions for expectant and new parents to be further explored.

The interpretation of this scoping review should consider its limitations. First, there is a possibility gray literature or important literature was missed written in language other than English, Dutch or French. Second, the included studies were conducted in the USA and India and therefore there might be cultural and socio-economic bias in this scoping review. Employment, child care and leave policies differ between countries and parts of the world which have affected findings in the included studies. Third, the quality of each study (i.e., methodology, design) included in this review was not assessed. Studies were included based on the study’s conclusions and implications of the intervention strategies on parental mental health.

## Conclusion and recommendations for research and practice

This scoping review has provided important insights and guidance for future research. The transition into parenthood of employed parents is complex and impacted by a variety of factors on different levels that encompass the parents’ life-context. There are still important gaps in the literature. Findings from this study may provide a roadmap for future research to close these gaps in knowledge. Therefore, we posit the following recommendations regarding future research:

- Interventions that promote mental resilience should include both *emotional and practical social support*, encompassing both the informal and work contexts;
- This scoping review also indicated an urgent need for *more in-depth research on intervention strategies for the group of employed expectant and new parents*. Future studies could best use a concrete *conceptualization of working parents’ transition to parenthood*, including the *social-cultural context* in which this transition occurs, the *nature of the work* parents perform (physical/emotional impact, uncertainty and developmental opportunities, and reward), and parents’ work characteristics (demand, stress, reward, autonomy);
- In addition, *qualitative (and mixed methods) research is needed in addition to quantitative research* to better understand parents’ barriers and facilitators in the transition to parenthood of *employed expectant and new parents*;
- There is currently a *lack of rigorous randomized control studies that evaluate specific interventions in terms of effectiveness*, as well as studies that test the implementation of such interventions in practice;
- Finally, future studies should include *vulnerable groups, such as parents with lower socioeconomic status or certain minority groups*, in order to better generalize research findings to the broader population.

## Supplementary Information

Supplementary Material 1.

Supplementary Material 2.

## Data Availability

Data is provided within the manuscript or supplementary information.

## References

[1] Salmela-Aro K, Nurmi JE, Saisto T, Halmesmaki E. Goal reconstruction and depressive symptoms during the transition to motherhood: evidence from two cross-lagged longitudinal studies. J Pers Soc Psychol. 2001;81(6):1144–59. 11761314 10.1037//0022-3514.81.6.1144

[2] The American College of Obstetricians and Gynecologists Committee Opinion no. 630. Screening for perinatal depression. Obstet Gynecol. 2015;125(5):1268-71. 10.1097/01.AOG.0000465192.34779. 10.1097/01.AOG.0000465192.34779.dc 25932866

[3] Seyfried LS, Marcus SM. Postpartum mood disorders. Int Rev Psychiatry. 2003;15(3):231–42. 15276962 10.1080/0954026031000136857

[4] Miller LJ. Postpartum depression. JAMA. 2002;287(6):762–5. 11851544 10.1001/jama.287.6.762

[5] Cox E. Women’s mood disorders a clinician’s guide to perinatal psychiatry. Chapel Hill: Springer; 2021.

[6] Lancet T. Women’s voices: speaking up about perinatal mental health. Lancet. 2017;389(10072):882. 10.1016/S0140-6736(17)30642-6 28271825

[7] O’Hara MW, Swain AM. Rates and risk of postpartum depression—a meta-analysis. Int Rev Psychiatry. 1996;8(1):37–54.

[8] Gavin NI, Gaynes BN, Lohr KN, Meltzer-Brody S, Gartlehner G, Swinson T. Perinatal depression: a systematic review of prevalence and incidence. Obstet Gynecol. 2005;106(5 Pt 1):1071–83. 16260528 10.1097/01.AOG.0000183597.31630.db

[9] Liu X, Wang S, Wang G. Prevalence and risk factors of postpartum depression in women: a systematic review and meta-analysis. J Clin Nurs. 2022;31(19–20):2665–77. 34750904 10.1111/jocn.16121

[10] Mitchell AR, Tong S, Hastie R. Estimated prevalence of perinatal depression in low- and middle-income countries considering uncertainty of test properties-reply. JAMA Psychiat. 2023;80(8):857. 10.1001/jamapsychiatry.2023.2019 37379012

[11] Garthus-Niegel S, Staudt A, Kinser P, Haga SM, Drozd F, Baumann S. Predictors and changes in paternal perinatal depression profiles-insights from the DREAM study. Front Psychiatry. 2020;11:563761. 33192683 10.3389/fpsyt.2020.563761 PMC7658469

[12] Thiel F, Pittelkow MM, Wittchen HU, Garthus-Niegel S. The relationship between paternal and maternal depression during the perinatal period: a systematic review and meta-analysis. Front Psychiatry. 2020;11:563287. 33192682 10.3389/fpsyt.2020.563287 PMC7658470

[13] Musser AK, Ahmed AH, Foli KJ, Coddington JA. Paternal postpartum depression: what health care providers should know. J Pediatr Health Care. 2013;27(6):479–85. 23182851 10.1016/j.pedhc.2012.10.001

[14] Smythe KL, Petersen I, Schartau P. Prevalence of perinatal depression and anxiety in both parents: a systematic review and meta-analysis. JAMA Netw Open. 2022;5(6):e2218969. 35749112 10.1001/jamanetworkopen.2022.18969 PMC9233234

[15] Dennis CL, Falah-Hassani K, Shiri R. Prevalence of antenatal and postnatal anxiety: systematic review and meta-analysis. Br J Psychiatry. 2017;210(5):315–23. 28302701 10.1192/bjp.bp.116.187179

[16] Hoffman C, Dunn DM, Njoroge WFM. Impact of postpartum mental illness upon infant development. Curr Psychiatry Rep. 2017;19(12):100. 29105008 10.1007/s11920-017-0857-8

[17] Sockol LE. A systematic review of the efficacy of cognitive behavioral therapy for treating and preventing perinatal depression. J Affect Disord. 2015;177:7–21. 25743368 10.1016/j.jad.2015.01.052

[18] Sockol LE. A systematic review and meta-analysis of interpersonal psychotherapy for perinatal women. J Affect Disord. 2018;232:316–28. 29501991 10.1016/j.jad.2018.01.018

[19] Evans K, Morrell CJ, Spiby H. Systematic review and meta-analysis of non-pharmacological interventions to reduce the symptoms of mild to moderate anxiety in pregnant women. J Adv Nurs. 2018;74(2):289–309. 28921612 10.1111/jan.13456

[20] Evans K, Spiby H, Morrell JC. Non-pharmacological interventions to reduce the symptoms of mild to moderate anxiety in pregnant women. A systematic review and narrative synthesis of women’s views on the acceptability of and satisfaction with interventions. Arch Womens Ment Health. 2020;23(1):11–28. 30613846 10.1007/s00737-018-0936-9 PMC6987064

[21] Missler M, Donker T, Beijers R, Ciharova M, Moyse C, de Vries R, et al. Universal prevention of distress aimed at pregnant women: a systematic review and meta-analysis of psychological interventions. BMC Pregnancy Childbirth. 2021;21(1):276. 33794828 10.1186/s12884-021-03752-2 PMC8017784

[22] Matvienko-Sikar K, Flannery C, Redsell S, Hayes C, Kearney PM, Huizink A. Effects of interventions for women and their partners to reduce or prevent stress and anxiety: a systematic review. Women Birth. 2021;34(2):e97–117. 32107141 10.1016/j.wombi.2020.02.010

[23] Verbeke E, Bogaerts A, Nuyts T, Crombag N, Luyten J. Cost-effectiveness of mental health interventions during and after pregnancy: A systematic review. Birth. 2022;49(3):364–402. 35322898 10.1111/birt.12623

[24] Spitzmueller C, Matthews RA. Work and the transition to motherhood: introduction. In: Spitzmueller C, Matthews RA, editors. Research perspectives on work and the transition to motherhood. Cham: Springer International Publishing; 2016. p. 1–8.

[25] Lucia-Casademunt AM, García-Cabrera AM, Padilla-Angulo L, Cuéllar-Molina D. Returning to work after childbirth in Europe: well-being, work-life balance, and the interplay of supervisor support. Front Psychol. 2018;9:68. 29467695 10.3389/fpsyg.2018.00068 PMC5808277

[26] Dagher RK, McGovern PM, Dowd BE. Maternity leave duration and postpartum mental and physical health: implications for leave policies. J Health Polit Policy Law. 2014;39(2):369–416. 24305845 10.1215/03616878-2416247

[27] Nichols MR, Roux GM. Maternal perspectives on postpartum return to the workplace. J Obstet Gynecol Neonatal Nurs. 2004;33(4):463–71. 15346672 10.1177/0884217504266909

[28] Dagher RK, McGovern PM, Alexander BH, Dowd BE, Ukestad LK, McCaffrey DJ. The psychosocial work environment and maternal postpartum depression. Int J Behav Med. 2009;16(4):339–46. 19288209 10.1007/s12529-008-9014-4

[29] Dagher RK, McGovern PM, Dowd BE, Lundberg U. Postpartum depressive symptoms and the combined load of paid and unpaid work: a longitudinal analysis. Int Arch Occup Environ Health. 2011;84(7):735–43. 21373878 10.1007/s00420-011-0626-7

[30] Schaber R, Karl M, Kopp M, Kress V, Weidner K, Martini J, et al. My job, my child, my house: the predictive value of job- and housework-related factors on depressive symptoms during the postpartum period. J Affect Disord. 2020;272:388–97. 32553382 10.1016/j.jad.2020.04.016

[31] Karl M, Schaber R, Kress V, Kopp M, Martini J, Weidner K, et al. Precarious working conditions and psychosocial work stress act as a risk factor for symptoms of postpartum depression during maternity leave: results from a longitudinal cohort study. BMC Public Health. 2020;20(1):1505. 33023543 10.1186/s12889-020-09573-w PMC7539402

[32] Waugh CE, Koster EH. A resilience framework for promoting stable remission from depression. Clin Psychol Rev. 2015;41:49–60. 24930712 10.1016/j.cpr.2014.05.004

[33] Van Haeken S, Braeken MAKA, Nuyts T, Franck E, Timmermans O, Bogaerts A. Perinatal resilience for the first 1,000 days of life. Concept analysis and Delphi survey. Front Psychol. 2020;11:563432. 33224056 10.3389/fpsyg.2020.563432 PMC7670043

[34] Sutton A, Clowes M, Preston L, Booth A. Meeting the review family: exploring review types and associated information retrieval requirements. Health Info Libr J. 2019;36(3):202–22. 31541534 10.1111/hir.12276

[35] Grant MJ, Booth A. A typology of reviews: an analysis of 14 review types and associated methodologies. Health Info Libr J. 2009;26(2):91–108. 19490148 10.1111/j.1471-1842.2009.00848.x

[36] Arksey H, O’Malley L. Scoping studies: towards a methodological framework. Int J Soc Res Methodol Theory Pract. 2005;8:19–32.

[37] Peters MDJ, Godfrey C, McInerney P, Munn Z, Tricco AC, Khalil H. Chapter 11: scoping reviews (2020 version). In: Aromatis e MZ, editor. JBI manual for evidence synthesis. JBI, 2020. 2020.

[38] Bramer WM, Giustini D, de Jonge GB, Holland L, Bekhuis T. De-duplication of database search results for systematic reviews in EndNote J. Med Libr Assoc. 2016;104(3):240–3. 10.3163/1536-5050.104.3.014 PMC4915647 27366130

[39] Ouzzani M, Hammady H, Fedorowicz Z, Elmagarmid A. Rayyan-a web and mobile app for systematic reviews. Syst Rev. 2016;5(1):210. 27919275 10.1186/s13643-016-0384-4 PMC5139140

[40] Tricco AC, Lillie E, Zarin W, O’Brien KK, Colquhoun H, Levac D, et al. PRISMA Extension for Scoping Reviews (PRISMA-ScR): checklist and explanation. Ann Intern Med. 2018;169(7):467–73. 30178033 10.7326/M18-0850

[41] Killien MG. The role of social support in facilitating postpartum women’s return to employment. J Obstet Gynecol Neonatal Nurs. 2005;34(5):639–46. 16227520 10.1177/0884217505280192

[42] Gjerdingen D, McGovern P, Attanasio L, Johnson PJ, Kozhimannil KB. Maternal depressive symptoms, employment, and social support. J Am Board Fam Med. 2014;27(1):87–96. 24390890 10.3122/jabfm.2014.01.130126 PMC3882899

[43] Jones KP, Brady JM, Lindsey AP, Cortina LM, Major CK. The interactive effects of coworker and supervisor support on prenatal stress and postpartum health: a time-lagged investigation. J Bus Psychol. 2022;37(3):469–90.

[44] Blewitt C, Savaglio M, Madden SK, Meechan D, O’Connor A, Skouteris H, et al. Using intervention mapping to develop a workplace digital health intervention for preconception, pregnant, and postpartum women: the Health in Planning, Pregnancy and Postpartum (HiPPP) portal. Int J Environ Res Public Health. 2022;19(22):15078. 36429795 10.3390/ijerph192215078 PMC9690929

[45] Costa J, Santos O, Virgolino A, Pereira ME, Stefanovska-Petkovska M, Silva H, Navarro-Costa P, Barbosa M, das Neves RC, Duarte E Silva I, Alarcão V, Vargas R, Heitor MJ. MAternal Mental Health in the WORKplace (MAMH@WORK): A Protocol for Promoting Perinatal Maternal Mental Health and Wellbeing. Int J Environ Res Public Health. 2021;18(5):2558. 10.3390/ijerph18052558. 10.3390/ijerph18052558 PMC7967657 33806518

[46] Ziaei S, Hammarstrom A. What social determinants outside paid work are related to development of mental health during life? An integrative review of results from the Northern Swedish Cohort. BMC Public Health. 2021;21(1):2190. 34847924 10.1186/s12889-021-12143-3 PMC8638423

[47] Fall A, Goulet L, Vezina M. Comparative study of major depressive symptoms among pregnant women by employment status. Springerplus. 2013;2(1):201. 23705107 10.1186/2193-1801-2-201 PMC3657078

[48] O’Hara MW. Social support, life events, and depression during pregnancy and the puerperium. Arch Gen Psychiatry. 1986;43(6):569–73. 3707289 10.1001/archpsyc.1986.01800060063008

[49] Herbell K, Zauszniewski JA. Stress experiences and mental health of pregnant women: the mediating role of social support. Issues Ment Health Nurs. 2019;40(7):613–20. 31021665 10.1080/01612840.2019.1565873

[50] Bedaso A, Adams J, Peng W, Sibbritt D. The relationship between social support and mental health problems during pregnancy: a systematic review and meta-analysis. Reprod Health. 2021;18(1):162. 34321040 10.1186/s12978-021-01209-5 PMC8320195

[51] Chojenta C, Loxton D, Lucke J. How do previous mental health, social support, and stressful life events contribute to postnatal depression in a representative sample of Australian women? J Midwifery Womens Health. 2012;57(2):145–50. 22432486 10.1111/j.1542-2011.2011.00140.x

[52] Xie RH, Yang J, Liao S, Xie H, Walker M, Wen SW. Prenatal family support, postnatal family support and postpartum depression. Aust N Z J Obstet Gynaecol. 2010;50(4):340–5. 20716261 10.1111/j.1479-828X.2010.01185.x

[53] Beck CT. Predictors of postpartum depression: an update. Nurs Res. 2001;50(5):275–85. 11570712 10.1097/00006199-200109000-00004

[54] Bernazzani O, Saucier JF, David H, Borgeat F. Psychosocial predictors of depressive symptomatology level in postpartum women. J Affect Disord. 1997;46(1):39–49. 9387085 10.1016/s0165-0327(97)00077-3

[55] O’Hara MW, Schlechte JA, Lewis DA, Wright EJ. Prospective study of postpartum blues. Biologic and psychosocial factors. Arch Gen Psychiatry. 1991;48(9):801–6. 1929770 10.1001/archpsyc.1991.01810330025004

[56] O’Hara MW. The nature of postpartum depressive disorders. Postpartum depression and child development. New York: Guilford Press; 1997. p. 3–31.

[57] Balaam MC, Kingdon C, Haith-Cooper M. A systematic review of perinatal social support interventions for Asylum-seeking and refugee women residing in Europe. J Immigr Minor Health. 2022;24(3):741–58. 34273047 10.1007/s10903-021-01242-3 PMC9072490

[58] Perry-Jenkins M, Laws HB, Sayer A, Newkirk K. Parents’ work and children’s development: a longitudinal investigation of working-class families. J Fam Psychol. 2020;34(3):257–68. 31414863 10.1037/fam0000580 PMC7021583

[59] Otto A, Bartova A, Van Lancker W. Measuring the generosity of parental leave policies. Soc Incl. 2021;9:238–49.

[60] Van Niel MS, Bhatia R, Riano NS, de Faria L, Catapano-Friedman L, Ravven S, et al. The impact of paid maternity leave on the mental and physical health of mothers and children: a review of the literature and policy implications. Harv Rev Psychiatry. 2020;28(2):113–26. 32134836 10.1097/HRP.0000000000000246

[61] Aitken Z, Garrett CC, Hewitt B, Keogh L, Hocking JS, Kavanagh AM. The maternal health outcomes of paid maternity leave: a systematic review. Soc Sci Med. 2015;130:32–41. 25680101 10.1016/j.socscimed.2015.02.001

[62] Heshmati A, Honkaniemi H, Juarez SP. The effect of parental leave on parents’ mental health: a systematic review. Lancet Public Health. 2023;8(1):e57–75. 36603912 10.1016/S2468-2667(22)00311-5

[63] Hidalgo-Padilla L, Toyama M, Zafra-Tanaka JH, Vives A, Diez-Canseco F. Association between maternity leave policies and postpartum depression: a systematic review. Arch Womens Ment Health. 2023;26(5):571–80. 37458837 10.1007/s00737-023-01350-z PMC10491689

[64] Whitney MD, Holbrook C, Alvarado L, Boyd S. Length of maternity leave impact on mental and physical health of mothers and infants, a systematic review and meta-analysis. Matern Child Health J. 2023;27(8):1308–23. 37043071 10.1007/s10995-022-03524-0

[65] Feldman R, Sussman AL, Zigler E. Parental leave and work adaptation at the transition to parenthood: individual, marital, and social correlates. J Appl Dev Psychol. 2004;25:459–79.

[66] Kornfeind KR, Sipsma HL. Exploring the link between maternity leave and postpartum depression. Womens Health Issues. 2018;28(4):321–6. 29729837 10.1016/j.whi.2018.03.008

[67] Whitehouse G, Romaniuk H, Lucas N, Nicholson J. Leave duration after childbirth: Impacts on maternal mental health, parenting, and couple relationships in Australian two-parent families. J Fam Issues. 2013;34:1356–78.

[68] Zhang C, Managi S. Functional social support and maternal stress: a study on the 2017 paid parental leave reform in Japan. Econ Anal Policy. 2020;65:153–72.

[69] Bilgrami A, Sinha K, Cutler H. The impact of introducing a national scheme for paid parental leave on maternal mental health outcomes. Health Econ. 2020;29(12):1657–81. 32935432 10.1002/hec.4164

[70] OECD Family Database. Available from: https://www.oecd.org/els/family/database.htm. Acessed 4 July 2022.

[71] Brym S, Mack JT, Weise V, Kopp M, Steudte-Schmiedgen S, Garthus-Niegel S. Mental health of working parents during the COVID-19 pandemic: can resilience buffer the impact of psychosocial work stress on depressive symptoms? BMC Public Health. 2022;22(1):2426. 36567325 10.1186/s12889-022-14582-y PMC9790816

[72] Engelhardt L, Mack J, Weise V, Kopp M, Starke KR, Garthus-Niegel S. The COVID-19 pandemic: implications for work-privacy-conflict and parent-child-bonding in mothers and fathers. Child Youth Serv Rev. 2023;155:107264.

[73] Koerber MI, Mack JT, Seefeld L, Kopp M, Weise V, Starke KR, et al. Psychosocial work stress and parent-child bonding during the COVID-19 pandemic: clarifying the role of parental symptoms of depression and aggressiveness. BMC Public Health. 2023;23(1):113. 36647046 10.1186/s12889-022-14759-5 PMC9841494

[74] Schaber R, Kopp M, Zähringer A, Mack JT, Kress V, Garthus-Niegel S. Paternal leave and father-infant bonding: findings from the population-based cohort study DREAM. Front Psychol. 2021;12:668028. 34149562 10.3389/fpsyg.2021.668028 PMC8212974

