# Supplementary material for: Intervention strategies to prevent mental health problems and improve resilience in employed parents from conception until the child is 5 years of age: a scoping review
Source: BMC Pregnancy Childbirth. 2025 Jan 9;25:17. doi: 10.1186/s12884-024-07043-4 (PMC11715540; doi:10.1186/s12884-024-07043-4)
Supplement: Supplementary file 1 — Supplementary Material 1. [file 12884_2024_7043_MOESM1_ESM.pdf]

Appendix I

#1	Perinatal	
	"Peripartum Period"[Mesh] OR "Postpartum Period"[Mesh] OR "Gravidity"[Mesh] OR "Pregnancy"[Mesh] OR perinatal[tiab] OR "Pregnant Women"[Mesh] OR prenatal[tiab] OR postnatal[tiab] OR postpartum[tiab] OR post-natal[tiab] OR post-partum[tiab] OR antenatal[tiab] OR pre-natal[tiab] OR ante-natal[tiab] OR peri-natal[tiab] OR peripartum[tiab] OR prepartum[tiab] OR pregnan*[tiab] OR gravidit*[tiab] OR gestation*[tiab] OR "early parenthood"[tiab] OR preconcept*[tiab] OR pre-concept*[tiab] OR puerperi*[tiab]	
#2	Parents	
	"Parents"[Mesh] OR maternal[tiab] OR paternal[tiab] OR maternity[tiab] OR paternity[tiab] OR "Parent-Child Relations"[Mesh] OR "Maternal-fetal Relations"[Mesh] OR "Mother-Child Relations"[Mesh] OR "Father-Child Relations"[Mesh] OR "Family"[Mesh] OR parent*[tiab] OR father*[tiab] OR mother*[tiab] OR mom[tiab] OR moms[tiab] OR mum[tiab] OR mums[tiab] OR momm*[tiab] OR mumm*[tiab] OR dad[tiab] OR dads[tiab] OR dadd*[tiab] OR "famil*"[tiab]	
#3	(mental) Health	
	"Stress, Psychological"[Mesh] OR stress*[tiab] OR distress*[tiab] OR "Burnout, professional"[Mesh] OR "burn out*"[tiab] OR burnout*[tiab] OR "burnt out"[tiab] OR "bore out*"[tiab] OR "bored out*"[tiab] OR "Occupational Stress"[Mesh:NoExp] OR "job stress"[tiab] OR "family stress"[tiab] OR "parental stress"[tiab] OR "family stress"[tiab] OR "parental stress"[tiab] OR "maternal stress"[tiab] OR "paternal stress"[tiab] OR "Stress Disorders, Post-Traumatic"[Mesh] OR "Psychological Trauma"[Mesh:NoExp] OR "Stress Disorders, Traumatic, Acute"[Mesh] OR trauma*[tiab] OR PTSD[tiab] OR "Anxiety"[Mesh:NoExp] OR anxiet*[tiab] OR anxious[tiab] OR "Depression"[Mesh:NoExp] OR depress*[tiab] OR "Psychotic Disorders"[Mesh:NoExp] OR psychos*[tiab] OR psychot*[tiab] OR "Depressive Disorder"[Mesh:NoExp] OR "Depression, Postpartum"[Mesh] OR wellbeing[tiab] OR "well-being"[tiab] OR "Mental health"[Mesh] OR "mental health"[tiab] OR "mental disease"[tiab] OR "Mood Disorders"[Mesh:NoExp] OR "mood disorder*"[tiab] OR "Resilience, Psychological"[Mesh] OR "Social vulnerability"[Mesh] OR "emotional vulnerab*"[tiab] OR "social vulnerab*"[tiab] OR resilienc*[tiab] OR "mental illness*"[tiab] OR "emotional disturbanc*"[tiab] OR "emotional distress"[tiab] OR "Mental Disorders"[Mesh:NoExp] OR "Maternal Behavior"[Mesh] OR "maternal behavio*"[tiab] OR "maternal care"[tiab] OR "paternal care"[tiab] OR "Paternal Behavior"[Mesh] OR "paternal behavio*"[tiab] OR "Maternal Welfare"[Mesh] OR "Maternal Health"[Mesh] OR "maternal health"[tiab] OR welfare*[tiab]	
	Employment	

#4	“Psychology, Industrial”[Mesh] OR “occupational psychology”[tiab] OR “industrial psychology”[tiab] OR workload[tiab] OR “boundary manag*”[tiab] OR “time manag*”[tiab] OR "Return to Work"[Mesh] OR career*[tiab] OR “Employment"[Mesh:NoExp] OR employ*[tiab] OR "Occupational Health"[Mesh] OR “occupational health”[tiab] OR “industrial health”[tiab] OR “Occupational Medicine”[Mesh] OR “occupational medicine”[tiab] OR “industrial medicine”[tiab] OR “Work”[Mesh:NoExp] OR “back to work”[tiab] OR “return to work”[tiab] OR job[tiab] OR jobs[tiab] OR “happiness at work”[tiab] OR “Work-Life Balance”[Mesh] OR “work-life balance”[tiab] OR “life work balance” OR “life work imbalance”[tiab] OR “work life imbalance”[tiab] OR “job satisfaction”[tiab] OR “jobsatisf*”[tiab] OR “jobdissatisf*”[tiab] OR “quality of working life”[tiab] OR “Work Performance”[Mesh] OR “work performance”[tiab] OR worker*[tiab] OR working[tiab]	
